# Role and Significance of Markers of Inflammation in the Asthmatic Disease

**DOI:** 10.3889/oamjms.2015.109

**Published:** 2015-10-21

**Authors:** Zlatica Goseva, Elena Jovanovska Janeva, Angelko Gjorcev, Zoran Arsovski, Sava Pejkovska

**Affiliations:** *PHI University Clinic of Pulmonology and Allergology, Faculty of Medicine, Ss Cyril and Methodius University of Skopje, Skopje, Republic of Macedonia*

**Keywords:** asthma, markers, inflammation, eosinophil, eosinophil cationic protein, IL-5

## Abstract

**BACKGROUND:**

Asthma is characterized by airway inflammation which can be reversible.

**AIM:**

Investigation the importance of eosinophils, ECP and IL-5 in asthmatics versus patients with obstructive bronchitis and healthy subjects. We investigated the values before and after the treatment in asthmatics.

**MATERIAL AND METHODS:**

We studied 77 subjects divided in three groups as follows: 1) asthma patients; 2) patients with obstructive bronchitis and 3) control group of healthy subjects. In all the subjects there were determined: Total number of eosinophils (Eo), eosinophilic cationic protein (ECP), Interleukin 5 (IL-5) and allergy tests.

**RESULTS:**

The total number of eosinophils was significantly increased in the group of asthma patients versus second and third group. We found that the presence of ECP demonstrate an ongoing inflammation, with or without clinical symptoms of asthma patients. There was significant difference between the values of ECP of asthma patients versus second and third group. Our results have shown that IL-5 was significantly increased versus second group and controls (p < 0.01). We also found the decrease of the values of inflammatory markers after the treatment with corticosteroids.

**CONCLUSIONS:**

Eosinophils, ECP and IL-5 could be useful markers for selecting allergic patients and could be the monitors of treatment effects.

## Introduction

The allergy is called an epidemic of the twentieth century. The allergic diseases are among the most common diseases in European countries and cover 15-30% of the (general) population. Asthma represents a possible disease of the entire human population and it is a rising illness. Worldwide about 2% of the total population suffers from asthma. Industrially developed countries: 2-10%; USA: approximately 18 million asthmatics, out of which about 4,8 are children; Russia: approximately 20 million inhabitants suffer from asthma; Republic of Macedonia: about 5% from the population (above 100 000).

The morbidity and the mortality caused by asthma continue to grow, even in the present, in times of advanced scientific researches and modern therapeutic treatment [1-5].

### Definition of asthma

Asthma is a heterogeneous disease, usually characterized by chronic airway inflammation. It is defined by the history of respiratory symptoms such as wheeze, shortness of breath, chest tightness and cough that vary over time and in intensity, together with variable expiratory airflow limitation. Both symptoms and airflow limitation characteristically vary over time and in intensity. These variations are often triggered by factors such as exercise, allergen or irritant exposure, change in weather, or viral respiratory infections. Symptoms and airflow limitation may resolve spontaneously or in response to medication, and may sometimes be absent for weeks or months at a time. On the other hand, patients can experience episodic flare-ups (exacerbations) of asthma that may be life-threatening and carry a significant burden to patients and the community [1].

### Ethyology of asthma

The most common asthmatics triggers are as follows: allergic (house dust, pollen, moled spores, fungi, animals, insects etc.); environmental factors (cold, moisture, smog etc.); professional irritants; respiratory infections; food and food additives; medicines (antibiotic, aspirin, analgesic etc.); hyperventilation and physical effort; vasculitis; parasitic infections and some other less expressed triggers.

There is also a genetic predisposition defined as atopy. If there is not atopic parent 15% of their children could be affected. If there is one atopic parent 40-50% of their children could be affected. If both parents are atopic 75% of their children could be affected.

Based on the ethiology asthma could be [1, 4, 5]: atopic, extrinsic - the exposure to certain allergens leads to inflammation of the airways, which results in asthmatic attacks; and nonatopic, intrinsic - an allergen is not identified.

### Pathogenesis of asthma

Pulmonary inflammation is characterized by: oedema, mucosal and vascular permeability, mucus secretion, reduction in mucociliary clearance, epithelial damage, subepithelial fibrosis, increased neuron sensitivity and cells infiltration.

T-cells are central to the immune response as *regulators* and *effectors* of the immune function. After chronic exposure to antigen, two subpopulations of T-cells may be identified as: T-helper1 (Th1) and T-helper2 (Th2). There is common participation with: B-lymphocyte cells, mast cells, eosinophils and macrophages. The atopic allergy is type-2 (Th2) hypersensitivity to antigen with genetic origin and environmental impact.

**Figure 1:**
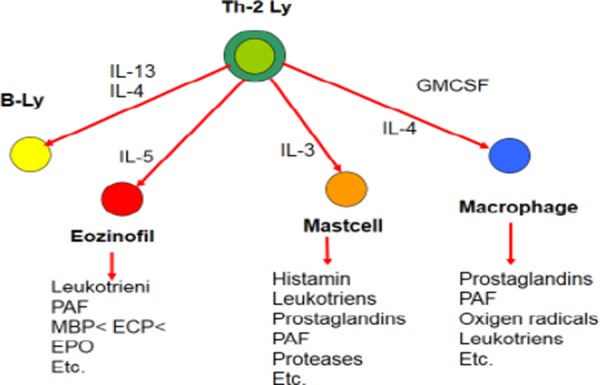
*Immunological reaction*.

Cytokines are low-molecular proteins which are produced by almost all eukaryotic cells and they are acting through specific “cell-surface” receptors. Cytokines have multiple activities, presented in the cytokine cascade. We can present the immunological reaction and cytokine cascade on Figure 1 [6].

**Figure 2:**
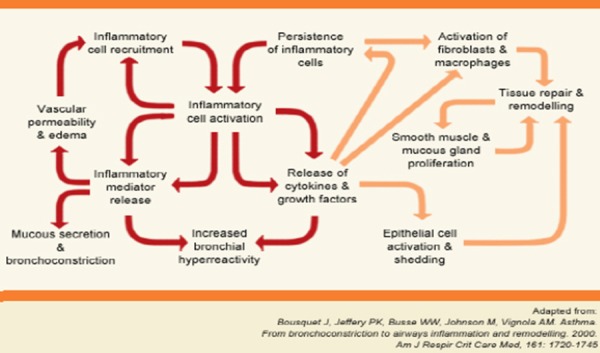
*Inflammation in airways*.

The inflammatory cells and various mediators cause damage of the airway (respiratory) epithelium, inducing bronchial hyperreactivity (Fig. 2) [7].

The bronchial obstruction is manifested in three stages [8]:

1) Bronchoconstriction (*early stage)* occurs 10-15 minutes after a spasmogenic contact with trigger stimulus. The stage is an early reaction with short term, with increased resistance in airways and it is reversible.
2) Second stage is bronchial obstruction with an inflammatory oedema of the bronchi, on which a mucus secret is complemented (related). It occurs 5-8 hours after the trigger contact; lasts a few days and it is sensitive to therapy with corticosteroids.
3) Subacute or chronic inflammation is severe inflammation expressed with infiltration and eosinophilic invasion.

Eosinophils are significant effector cells, involved into the late and chronic stage of the allergic inflammatory response. The tissue eosinophilia is mainly a result of the cured IL-5 of the allergen specific T-Ly [9-11].

According to literature, the values of ECP are [11, 12]: higher among atopic people 48-72 hours after allergenic exposition; higher during the pollen season; correlate with Eosinophils (Eo); correlate with the severity of asthma; more useful for chronic inflammation; are not specific for asthma; higher values at rhinitis or eczema (non-asthmatic atopic people); and significant for monitoring of the treatment.

Interleukin 5 (IL-5) has the central part in the maturation of eosinophils (Eo), with their release, activation, extension of survival and degranulation [10, 11].

The aim of our study was to show the importance of inflammation as key of asthma; to determine some parameters as markers of the inflammatory process; to show the significance of Eo and ECP, while measuring the level of a specific type of inflammation; to make an investigation of ECP as a marker of antigen exposition; to make an investigation if the value of ECP could be an indication of the effect of the treatment and to determine the significance of IL-5 in the inflammatory chain and the evolution of asthma.

## Material and Methods

We have investigated 77 subjects at the age among 23 and 79 years old. At each subject, there were complete examinations made for proper diagnosis and categorization.

### Groups of the patients

1^st^ group: 44 patients with asthma (15 with intrinsic asthma and 29 with extrinsic asthma);

2^nd^ group: 21 patients with chronic obstructive bronchitis;

3^rd^ group: 12 healthy subjects.

The classification was according to the actual version of the GINA guidelines (Global Initiative of Asthma) [1] and actual version of Global Initiative for Chronic Obstructive Lung Disease (GOLD) [13].

In all subjects we were investigated: total number of eosinophils (Eo); eosinophilic cationic protein (ECP); Interleukin 5 (IL-5) and allergy skin tests.

### Statistical analysis

The results were statistically analysed according to the ANOVA test, Kruskal-Wallis test, Wilcoxon and Man Whithey test. The significances value were taken p<0.05 and a highly significant p < 0.01.

## Results

**Figure 3:**
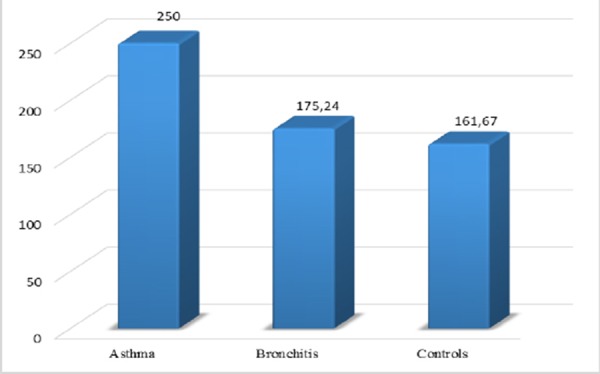
*Average values of the total number of Eo among the examined groups. ANOVA: Fx = 13.099, DF = 2, p < 0.01*.

In our study there is significant deference between the average values of Eo in asthmatics versus average values of Eo in patient with obstructive bronchitis and healthy controls (Fig. 3).

**Figure 4:**
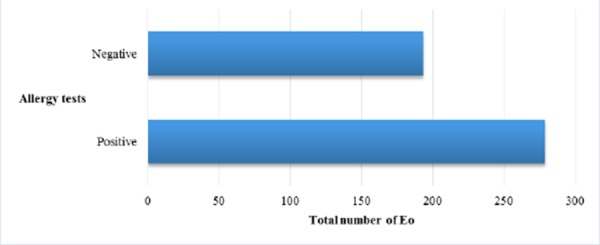
*Comparison of Eo values between intrinsic and extrinsic asthma. Mann-Whithey test: p < 0.01*.

Total number of eosinophils was significantly increased in patient with intrinsic versus extrinsic asthma (Fig. 4).

**Figure 5:**
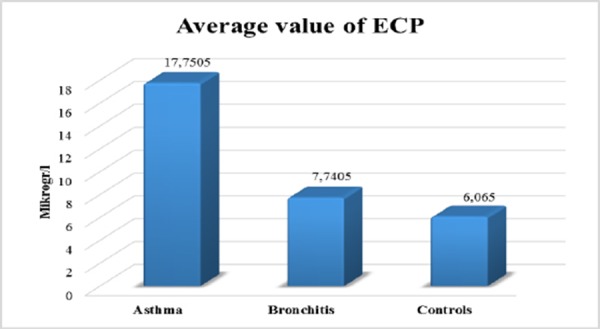
*Average value of ECP. Kruskal Wallisov test Hi = 16.579, DF = 2, p < 0.01*.

Average values of ECP were significantly increased in asthmatics versus ECP in patient with obstructive bronchitis and group of healthy controls (Fig. 5).

**Figure 6:**
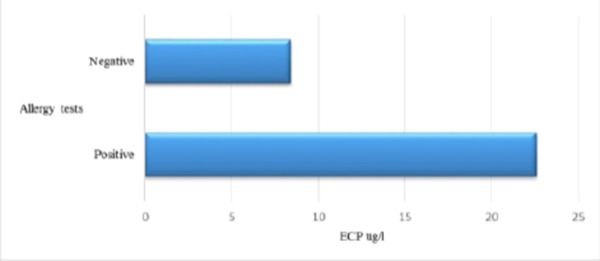
*Comparison of ECP values between intrinsic and extrinsic asthma. Mann-Whithey test: p < 0.01*.

Values of ECP were significantly increased in patients with intrinsic versus extrinsic asthma (Fig. 6).

**Figure 7:**
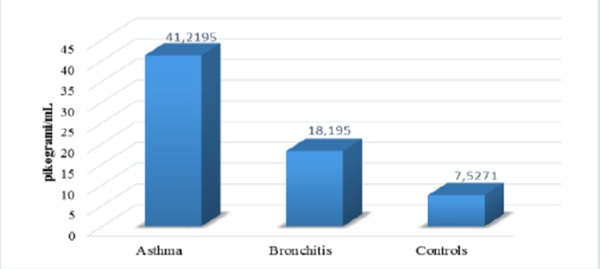
*Average values of IL-5. Kruskal Wallisov test Hi = 21.958, DF = 2, p < 0.01*.

Average values of IL-5 were significantly increased in asthmatics versus IL-5 in the group of patients with obstructive bronchitis and group of healthy controls (Fig. 7).

**Figure 8:**
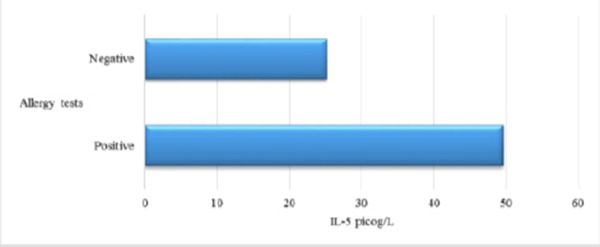
*Comparison of IL-5 values between intrinsic and extrinsic asthma. Mann-Whithey test: p < 0.05*.

Values of IL-5 were significantly increased in patients with intrinsic versus extrinsic asthma (Fig. 8).

**Figure 9:**
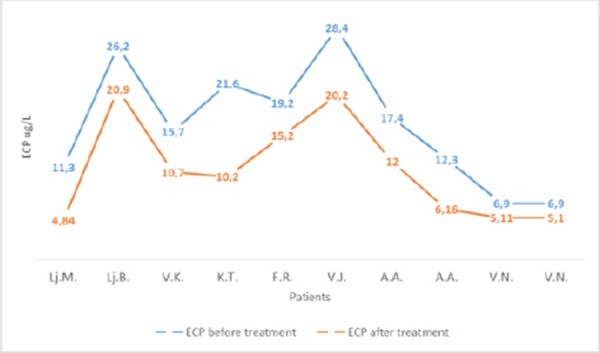
*Values of ECP in patients with asthma before and the after treatment with corticosteroids. Wilcoxon test p < 0.01*.

The values of ECP in patients with asthma were significantly decreased after treatment with corticosteroids (Fig. 9).

The values of IL-5 in patients with asthma were significantly decreased after treatment with corticosteroids (Fig. 10).

**Graphic 8:**
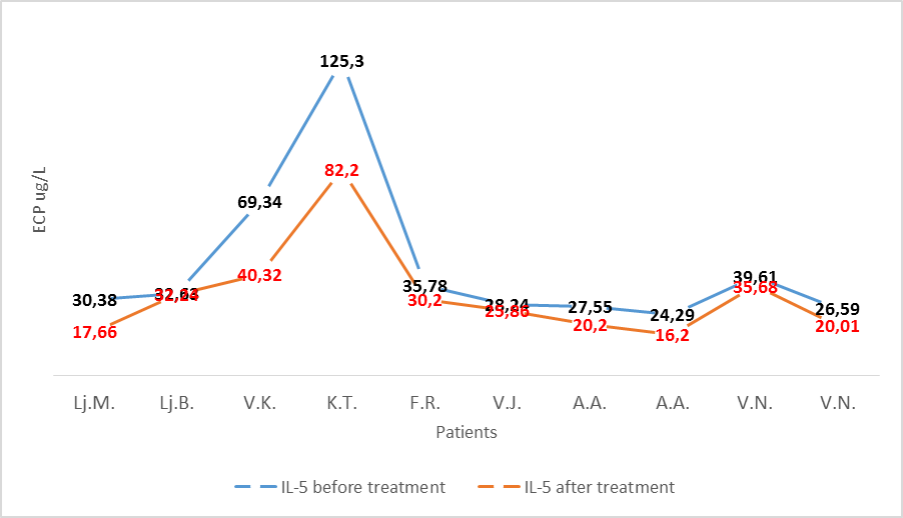
*Values of IL-5 in patients with asthma before and the after treatment with corticosteroids. Wilcoxon test p<0.01*.

## Discussion

Activated Eo in asthmatics delivered granulated proteins, like ECP detected in bronchial biopsies, BAL, sputum and blood (De Blay F. et al) [16]. According to our results there is significant deference between the average values of Eo and ECP in asthmatics versus average values in patient with bronchitis and healthy controls. Bousquet in a study tells that in cases with COPD the values of ECP are not raised [14].

We noticed significant correlation between the values of ECP and skin allergy tests as it is known in literature. Our results did not show significant correlation between ECP, FEV1 and symptom score. According Ronchi serum ECP values were increased in asthmatics versus healthy controls, but there was not the correlation with clinical symptoms and pulmonary function [19]. In our results we found positive correlation between ECP and total number of Eo. The study of Vaterlla says that the value of ECP is significantly increased during pollen season and the changes of ECP significant correlated with the changes of total number of Eo [20]. Increased values of ECP in sera could suggest asthma, but they are not specific for asthma as ECP could be increased in non-asthmatic patients with rhinitis, eczema, urticarial etc.

There is significant deference between the values of IL-5 in asthmatics versus group of patients with bronchitis and healthy controls. IL-5 suggests inflammation in asthma [17, 18]. There is positive correlation between values of IL-5 and allergy tests. Japanese authors in their study concluded that the production of IL-5 is higher in asthmatics then in healthy subjects as follows: atopic asthma – 37.6 ± 7.1 pg/ml; non-atopic asthma – 25.7 ± 5.5 pg/ml and halthy subjects – 3.0 ± 0.4 pg/ml [22]. There is not fowned positive correlation between the values of IL-5 and symptom score. The explination could be in the opinion that IL-5 is cytokine of late phase of the reponse and it is not detectable very soon after antigen provocation.

The values of ECP and IL-5 have tendetion of decrease after corticosteroid therapy. In literture there are several studies according which anty-inflammatory therapy decrease the values of the markers as ECP and IL-5.

In conclusion, the investigations that were made, confirm the concept that asthma is a chronic inflammatory disease. Determining and measurements of the markers of inflammation contributes to the diagnosis of asthma, monitoring the course of disease and contribute in the proper treatment of the asthmatic patients.

There is significant difference of the values of the eosinophils in asthmatics versus patients with bronchitis and healthy subjects. Total number of eosinophils versus patients with obstructive bronchitis is not different as in healthy controls.

There are significantly higher values of ECP in asthmatics versus patients with obstructive bronchitis and healthy subjects. Correlation between ECP and FEV1 and ECP and symptom score were not found. ECP could be higher in non-asthmatic atopic patients like patients with rhinitis, egzema, urticaria. There is significance in average values of IL-5 in asthmatics versus values of IL-5 in patients with obstructive bronchitis and healthy subjects. We found positive correlation between Eo and ECP and Eo, ECP and IL-5 with allergy tests. Values of ECP and IL-5 decreased after therapy with corticosteroids.

Values of markers of inflammation have their importance together with other tests ad criteria for proper diagnosis and treatment of asthma.
